# Effect of evidence-based approach on the customer orientation (Case study: Physicians Health Centers in Isfahan province in 2014)

**Published:** 2015

**Authors:** NG Esfahani, Y Maharati

**Affiliations:** * Health vice Chancellery of Isfahan Medical University, Isfahan University of Medical Sciences, Isfahan, Iran,; ** Faculty of Economic and Administrative Sciences, Ferdowsi University of Mashhad, Mashhad, Iran

**Keywords:** approach based on evidence, customer orientation, knowledge management, medicine based on evidence

## Abstract

**Introduction:** The purpose of this research was to examine the approach, based on evidence to customer-orientation attending physicians in the region, them being the subject collection.

**Research method:** This is a definitive-analytic and cross-sectional configuration, which was completed in 2014. The statistical community in this research consists of 212 doctors in the healthcare hubs. The working physicians chose 200 patients by means of a simple accidental sampling. The analysis means was the researcher built survey whose efficacy and reliability were verified. In this research, the fundamental equation design and partial least square technique were applied to examine the presumptions and fitness pattern and the structural design was agreed as adequate.

**Findings:** The outcomes showed four cases linked to the character, a behavior which was meant to treat; traditional origins of evidence were employed to retrieve data based on the reliable evidence and the shortage of limitations to the performance of client orientation strategy of evidence-based influences were meaningful. Two ranges of the doctor's information, the absence of restrictions, and the finding of the sign of dimensions about the client orientation approach were according to the evidence that had no meaningful influence.

**Conclusion:** The utilization of evidence-based training not only increased awareness, character, and abilities of the doctors but also allowed them to answer to the requirements of clients in choosing excellent decisions and presenting a better quality of healthcare, by decreasing treatment prices for patients, bringing satisfaction of patients, and finally having a better effectiveness for patients and institutions.

## Introduction

Since the first contact doctors are in health centers, therefore, patients in public centers are considered one of the most important groups in providing a key role in the use of the evidence-based approach in their daily activities and clinical decision-making [**1**]. Combining the best scientific evidence, needs and values, the environment, corporate resources, as well as human resources, can provide appropriate evidence-based decisions [**2**]. The job of the employees’ is the involvement and training in providing effective aids to the requirements of the customer satisfaction [**3**].

**Theoretical literature research:**

**A. Evidence Based practice:** Evidence can be information or facts that show the definition of default or a belief that is true, valid, or not valid (Concise Oxford). The amount of funds required for the regional evidence is relevant and specific issues should also be. UNFPA is an evidence-based approach that is defined as a “systematic effort to provide the best empirical evidence in decision-making for planning, implementation, and monitoring and evaluation program” [**4**]. David Lawrence Sackett explained evidence-based medication as an integrating clinical ability with the greatest clinical evidence from systematic research available to the best possible management of foreign patients [**5**]. Firstly, the need to learn the skills of **E**vidence-**B**ased **M**edicine **(EBM)** is represented by a very high volume of medical information and is rapidly increasing. Secondly, physicians may need less time to devote to study. In addition, studies showed that the efficiency workshops are common, so they need to learn methods in order to continue education during lifetime [**6**].

**B. Customer orientation:** among the competition in the international arena and in this period, the correct application of sources is considered as one of the greatly challenging elements of management and the connection among the employees and customers are seen as one of the institution's resources. The organizations should try new concepts of modern marketing, which means the art of finding, keeping and developing customer needs and new demands created for customers in order to bypass the exercise of power, but also using participation and understanding to manage relationships with customers and provide them to ensure their profitability [**7**]. Managers should find the time and tools available to their employees to an evidence-based approach in their daily activities application. Managers should be able to structure and culture the evidence-based practice in their own organization [**8**]. Customer relationship management by using information technology and organizational changes tries to re-engineer processes and turn them into customer-centric processes [**9**].

**C.** The relationship between the evidence-based practice and the customer orientation on knowledge management strategy, total quality management and customer focus have a special place. The purpose of this strategy is to achieve a superior service that seeks to create a culture of customer orientation with the identification of the needs and expectations of customers and measure their satisfaction to the knowledge, attitudes, skills and behaviors that the employees nurture to achieve its ultimate goal of customer orientation [**10**].

By providing the necessary resources and evidence, removing barriers to employment, promoting the culture of participation and sharing of staff, staff training encourages and motivates doctors to switch to the use of **EBM**, to take the appropriate decisions in the treatment; the same customer satisfaction being the ultimate goal which can be achieved. Therefore, the main hypothesis of this study was determined: an evidence-based approach of the doctors on the effective customer orientation.

**History of research**

The outcomes of the research of Heiwe et al. in the field of attitudes, knowledge and behavior of health care professionals, showed that the groups have a positive attitude towards the evidence-based practice in their decision making and clinical work. The majority of them had the power to analyze and evaluate the existing evidence, guidelines, and instructions that were available. Most of the obstacles regarding their performance problems were expressed. Finally, the researcher supported the Chief Executive Officer concerning the factors affecting the evidence-based performance, evidence-based and outcome performance and gratification of patients and contributed to productivity [**11**].

In a qualitative research conducted in the field of evidence-based understanding of nurses, it was achieved that the evidence from the research was used in nursing. Nurses needed to recognize the value and significance of the research and the application of its results was difficult, the addition to the emphasis on the concept of evidence-based care being suggested, also the production of methods, recovery and the evaluation of research evidence in nursing education programs. Incentive policies for nurses with evidence-based practice, the improvement of their knowledge, skills, and the quality of attention should also be considered [**12**].

In a study of Kermanshahi and Parvinian, the nurses’ views on barriers to implementing evidence-based care were examined and the outcomes revealed that nurses had barriers in implementing the evidence-based care for the management and included the insufficient number of staff and managers’ knowledge of the value of evidence-based care. In the personal-care dimension, the shortage of enough time of nurses to examine the research was one of the very significant barriers [**13**].

The outcomes of the research of Salehi and Abedi regarding the implementation of evidence-based performance on the nurses explained that in terms of achievement in the area of evidence-based nursing, many of the staff working in this area was weak, the researchers were motivated and the organization supported this weakness [**14**].

In a semi-experimental study entitled “The impact of evidence-based clinical training on the quality of the patient care and satisfaction”. It was found that the evidence-based education was applied to promote knowledge, skills and enhance the condition of patient care [**15**].

There are a few studies performed on the physicians’ knowledge about EBM in the Middle East. In 2004, a study of AL Baghil and AL Almaie showed that only 40 percent of Saudi Arabia primary health care physicians have learnt something regarding **EBM** [**16**].

In another study conducted in the UK, it was reported that 40% of the general physicians had knowledge about the search methods based on evidence and 71 percent of the time, they lacked the most significant portion in the decision of not having to use evidence-based medicine [**17**].

Other studies showed that physicians need a clear understanding of the terms used in evidence-based medicine. In a study in the field of awareness and the use of evidence-based medicine among residents of Shiraz University of Medical Sciences conducted by Amini et al., it was shown that residents with positive attitudes toward medicine based on evidence and the access to Internet for clinical decision making, practically did not use evidence-based medicine and were unaware of specific websites. The reason may be that they were not trained in this field [**18**].

A research of Dalheim et al. on the factors affecting the improvement of evidence-based training of working nurses concluded that the colleagues' and nurses experience was used in evidence-based practice. However, because of the obstacles, evidence from research was rarely used. The most important obstacles in Evidence-Based Practice lacked time and abilities to search, records management, research, nursing age, and a quantity of years as far as the working nurses were concerned [**19**].

In a study of MacDermid and Graham on a group of practicing midwifery profession after a period of training in EBP, it was found that participants in the study were extremely excited regarding the EBP, at the same time believing that this approach enhanced the critical thinking skills, increased confidence and a better care of the patients [**20**].

In a study of Morris and Maynard in the field of evidence-based care in midwifery, it was proved that the overall objective was the one of empowering the evidence-based care in identifying and understanding the needs of patients, clients, and midwifery practitioners, in decision-making and the application of scientific findings in the final midwifery care [**21**].

**Table 1:** Benefits of using evidence-based practice based on qualitative and quantitative results of studies from 2000 to 2010

Evidence-Based practice Benefits	Row
Improving the quality of aids and health care and finding the need [**4**-**22**]	1
Increasing public participation and teamwork and brainstorming skills among staff [**3**-**25**]	2
In response to the client’s decision-making skills and power to serve the needs of clients and evidence into the best performance [**3**-**22**]	3
Reduce the gap between theory courses passed in the university and practical work [**23**]	4
Increase the power of critical thinking and problem-solving skills and services (knowledge, skills and performance) [**3**-**22**]	5
Reduce the cost of treatment, reduce the length of hospital stay (time management) [**4**,**26**]	6
Increased patient satisfaction regarding the care [**24**]	7
Increased sense of confidence and flexibility in staff [**25**,**27**]	8
Increase accountability of employees [**22**,**24**,**26**]	9
Increase learning skills, use of technology, especially the usage of the Internet [**22**,**28**]	10
Skills increase training and transfer of scientific information to clients [**23**]	11

According to rows 1,3,6,7 and 11 in **Table 1**, it can be said that in previous investigations, the connection between evidence-based and customer-oriented approach was proven, but none of them specifically investigated the linkage among these two variables. Therefore, we considered the causal linkage among the two variables in this research.

**Research hypotheses**

**Hypothesis 1:** Attitudes of physicians in the area of evidence-based approach, which have a meaningful positive influence on client satisfaction.

**Hypothesis 2:** The behavior of physicians based on evidence-based approach, which has a meaningful positive influence on client satisfaction.

**Hypothesis 3:** The lack of barriers in investigating and finding evidence of a meaningful positive influence on client satisfaction. 

**Hypothesis 4:** Getting familiar with the evidence-based approach to customer focus and a meaningful positive influence. 

**Hypothesis 5:** Lack of barriers in the achievement of the greatest evidence of a significant positive effect on customer satisfaction. 

**Hypothesis 6:** Sources of evidence commonly used to retrieve customer knowledge have a significant positive influence. 

**Conceptual research model:**

**Fig. 1:**
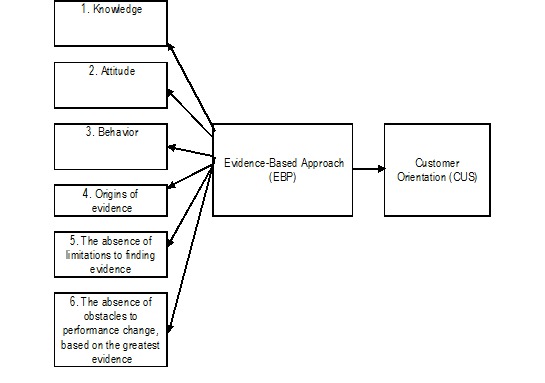
Effect of an evidence-based strategy to customer service (Case study: medical health centers in Isfahan)

## Research method

In titles of nature and purpose, the current study is functional and in titles of the method of data collection, it represents a descriptive survey of the connection between the causal variables. Doctors operating in the healthcare centers of the province 1 and 2 represented the statistical society examined in this research. The sample was random, the sampling system giving the minimum requirements for 200 physicians working in health centers 1 and 2 of Isfahan province. 200 questionnaires were distributed between respondents and the same 200 questionnaires were suitable for analysis, identifying a numerous statistical analysis and evidence-based practice to estimate the customer satisfaction questionnaire prepared. The first part of the application included demographic features and the another part evidence-based practice, which consisted of 30 questions. The evidence-based practice consisted of six levels, attitudes and behavioral intention (5 items), behavior of physicians on evidence-based practice (5 items), shortage of barriers regarding the performance based on the greatest evidence (4 items), knowledge (4 items), lack of barriers regarding investigation and evidence (5 questions), origins of evidence common for data recovery (4 items). The third section of the questionnaire was relevant to customer questions (3 items) [**1**-**30**].

In **Table 2**, the number of measures designed to measure variables, Cronbach’s alpha coefficient, and reliability of combined variables were presented.

**Table 2:** Cronbach’s alpha coefficient and reliability of combined research variables

Reliability	Cronbach alpha	code	Variable
0.909	0.875	ATI	Attitude toward evidence-based practice
0.785	0.676	BHV	Behavioral intention and behavior
0.799	0.663	EVI	No obstacles regarding the investigation and evidence
0.821	0.736	KNO	Introduction to Evidence-Based Practice
0.818	0.717	PER	No barriers related to vary based on the greatest evidence
0.761	0.616	RES	Evidence sources commonly used for data recovery
0.713	0.604	CUS	Customer Orientation

As it could be observed from all the variables in this research, Cronbach's alpha coefficient was of at least 0.6, 0.65, remarkably higher. To assess the validity (convergent), the exploratory factor analysis was used for factor analysis, index KMO, Bartlett test, and convergent validity [**4**-**32**].

**Table 3:** Number of measures designed to evaluate the soundness of each variable

Sampling criteria KMO	Approximate value 2χ	Freedom degree	Bartlet significance	Factorial loading	Items	Name of variable
0.837	545.506	15	0.000	0.674	ATI1	Attitude toward evidence-based practice
				0.634	ATI2	
				0.784	ATI3	
				0.692	ATI4	
				0.520	ATI5	
0.676	212.308	15	0.000	0.3	BHV1	Behavioral intention and behavior
				0.350	BHV2	
				0.579	BHV4	
				0.454	BHV5	
				0.408	BHV6	
0.613	210.630	6	0.000	0.662	PER1	Lack of barrier relates to vary based on the greatest evidence
				0.661	PER2	
				0.482	PER3	
				0.377	PER4	
0.664	210.226	6	0.000	0.375	KNO1	Introduction to Evidence-Based Practice
				0.597	KNO2	
				0.602	KNO3	
				0.678	KNO4	
				0.330	EVI2	
				0.425	EVI3	
				0.506	EVI4	
				0.353	EVI5	
				0.464	EVI6	
0.609	264.172	28	0.000	0.481	RES1	Evidence sources commonly used for data recovery
				0.426	RES2	
				0.456	RES4	
				0.335	RES5	
0.553	21.386	3	0.000	0.373	CUS1	Customer Orientation
				0.581	CUS2	
				0.421	CUS3	

Bartlett and KMO test results showed that the index values were desirable. KMO standard variable rate of larger than 0.5 and smaller than 0.05 for the CLS was also determined by Bartlett test. Items that amounted smaller than 0.03 and which were not compatible with other items were dismissed from the analysis. To check the soundness of the (credit) converge in the PLS model, the mean-variance extracted (AVE) was used. As it can be seen in **Table 4** below, all the average variance obtained was of more than 0.5, therefore, an appropriate model of convergent validity was highlighted [**33**-**35**].

**Table 4:** Convergent validity of the constructs of research variables

RES	PER	KNO	EVI	CUS	BHV	ATI	Convergent validity \ validity
0.654	0.637	0.548	0.502	0.557	0.532	0.667	Average of obtained variance (AVE)

To assess the reliability of any of the markers in the latent variable PLS model, the load factor of each indicator was determined. The value of each hidden variable load factor markers had to be larger than or equivalent to 3.0. 

**Table 5:** The value of latent variables load factor markers

P values	CUS	RES	PER	KNO	EVI	BHV	ATI	Variable \ marker	Row
<0.05							0.840	ATI1	1
<0.05							0.797	ATI2	2
<0.05							0.904	ATI3	3
<0.05							0.824	ATI4	4
<0.05							0.707	ATI5	5
<0.05						0.477		BHV1	6
<0.005						0.543		BHV2	7
<0.005						0.844		BHV4	8
<0.005						0.693		BHV5	9
<0.005						0.666		BHV6	10
<0.05					0.625			EVI2	11
<0.05					0.796			EVI3	12
<0.05					0.775			EVI4	13
<0.05					0.620			EVI6	14
<0.05				0.752				KNO1	15
<0.05				0.854				KNO2	16
<0.05				0.622				KNO3	17
<0.05				0.684				KNO4	18
<0.05			0.882					PER1	19
<0.05			0.818					PER2	20
<0.05			0.610					PER3	21
<0.05			0.576					PER4	22
<0.05		0.774						RES1	23
<0.05		0.6						RES2	24
<0.05		0.801						RES4	25
<0.05		0.465						RES5	26
<0.05	0.555							CUS1	27
<0.05	0.758							CUS2	28
<0.05	0.697							CUS3	29

As it could be seen in the above table, all the measures linked to the underlying variable that housed more than 40%, were marked as shaded. Therefore, the model could measure latent variables that were indicators of reliability in the field. All the amounts of the indicators were expected to be less than 0.05 and the soundness of research tools were appropriate.

**Findings**

**Fig. 2:**
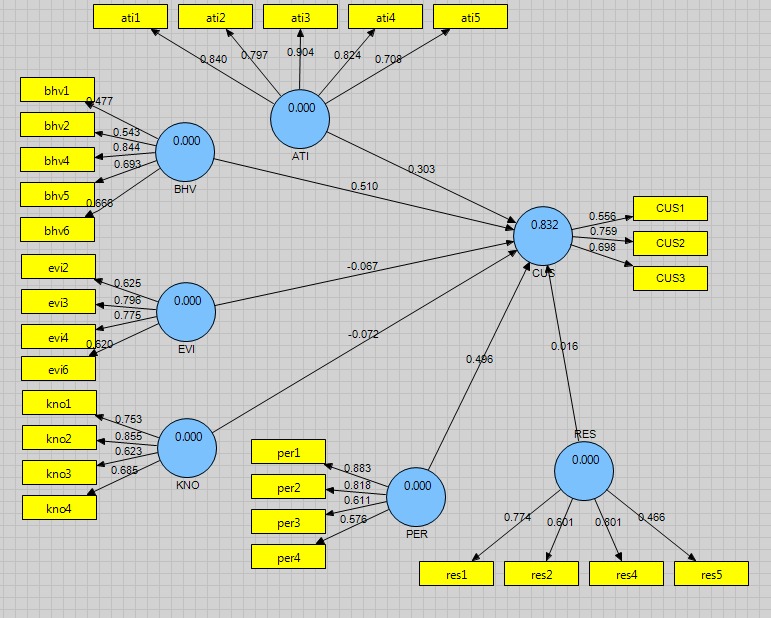
The research model in the standard estimate

100 respondents in the Health Center of Isfahan city [**1**] and 100 [**2**] of the city health center were employed (95 males and 105 females). 193 respondents’ education level was Ph.D. and 7 were experts. Regarding the position, the organization was responsible for 63 health centers, 120 healthcare centers, and 17 family physicians. **Fig. 2** revealed the connection between the 2 variables, the path coefficient of performance evidence-based approach and customer of 0.303 respectively.

**Fig. 3:**
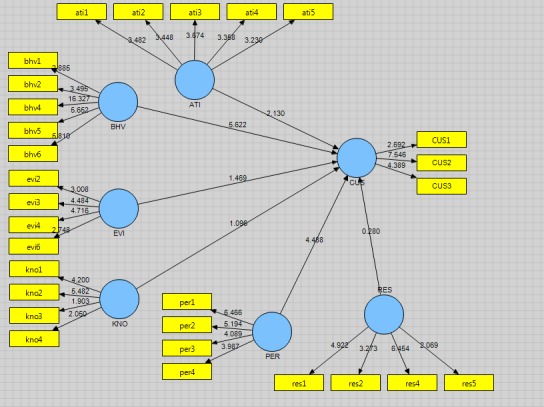
The research model in significant mode parameters

Given the probability (p-value), it had a less significant level of 0.05. In fact, a significant number was out of range (1.96, -1.96) (**Fig. 3**). It can be achieved that the path coefficients were significant, at a significant level of 0.05, meaning that the approach of evidence-based practice had a notable influence on customer satisfaction. With respect to the second hypothesis of the research, the coefficient of relationship between the two variables of tracking customer behavior and behavioral intention was calculated to be of 0.510 (**Fig. 1**).

Given the probability (p-value), it had a less significant level of 0.05. In fact, a significant number was out of range (1.96, -1.96) (**Fig. 3**). It can be concluded that the path coefficients were significant, at a significant level of 0.05, meaning that the purpose and attitude of the customer impact were significant. In connection with the third hypothesis study, the coefficient of the relationship was computed between the two variables, no obstacles being encountered on the path of finding evidence and customer orientation and being of -0.067 (**Fig. 2**).

Given the probability (p-value), it had a significant level of more than 0.05. In fact, a significant number was in the range (1.96, -1.96) (**Fig. 3**). It can be achieved that this was not a significant factor (0.05) path error, meaning the shortage of barriers regarding the investigation and finding no evidence of a significant impact on customer orientation. In connection with the fourth research hypothesis, the path coefficient was computed for the connection among two variables Introduction to Evidence Based Practice and customer orientation and its value was -0.072 (**Fig. 2**).

Given the probability (p-value), it had a significant level of more than 0.05. In fact, a significant number was in the range (1.96, -1.96) (**Fig. 2**). It can be concluded that this was not a significant factor (0.05) path at a significant level; meaning that the familiarity with the practice of evidence-based customer orientation did not have a meaningful influence. In connection with the fifth research hypothesis, the path coefficient for the connection among two variables was based on the best evidence and the shortage of barriers to changing customer orientation, being of 0.496 (**Fig. 2**).

Given the probability (p-value), it had a less significant level of 0.05. In fact, a significant number was out of range (1.96, -1.96) (**Fig. 3**). It can be concluded that the path coefficients were significant, having errors of 0.05; meaning a shortage of barriers in investigating and finding evidence of a significant impact on customer orientation. In connection with the sixth research hypothesis, the path coefficient for the connection within two variables common sources of evidence used to retrieve customer orientation information, was 0.016 [**2**].

Given the probability (p-value), it had a significant level of more than 0.05. In fact, a significant number was out of range (1.96, -1.96) (**Fig. 3**). It can be concluded that the path coefficients were significant; having errors of 0.05, indicating that the most frequent sources of evidence used to retrieve customer orientation data had a significant influence.

**Assessment of the indices model fitting**

To check the quality or reliability of the pattern, which included a credit check share index and index credit check, redundancy was used. In **Table 6**, the values of any of the indicators of the independent variables were affiliated mediators. As it can be viewed, the indicators were positive and higher than zero.

**Table 6:** Share indices (CV Com) and redundant index (CV Red)

CV Red	CV Com	Variable
0.504	0.504	Attitude toward evidence-based practice
0.174	0.174	Behavioral intention and behavior
0.185	0.185	No obstacles to the investigation and evidence
0.247	0.247	Introduction to Evidence Based Practice
0.265	0.265	No barriers related to change based on the best evidence
0.135	0.135	Evidence sources commonly used for data recovery
0.376	0.035	Customer orientation

## Discussion and Conclusion

According to the outcomes of the research, a hypothesis of a causal connection between the attitudes of the physicians on evidence-based exercise was accepted by the customer orientation. The study results showed that physicians have a positive attitude towards evidence-based exercise and believe in the physicians’ submission of evidence-based practice in high-quality services to provide better and faster health services, giving the best response to the requirements of the recipients of health care services and satisfaction customer orientation impact that made up the health care system. The outcomes of the previous study also indirectly suggested that this approach was based on evidence-based practice to have an influence on the customer orientation [**11**,**18**].

According to the second hypothesis regarding the impact of behavioral intention, the behavior based on evidence-based practice to customer orientation was evident, the physicians were studied, and the behaviors were associated with evidence-based practice in order to give the best answer to the clients. These behaviors included problem-solving skills, filling gaps in professional performance by using the best evidence, using guidelines and instructions in response to the client needs and skills of the professional practice due to new evidence. As a consequence, the number of previous researches also indirectly referred to the issue of the treatment based on evidence-based practice which affected the customer orientation [**11**,**15**].

Amini and colleagues research results were different from the conclusions of the current study, and, the researchers concluded that the residents in the research had a positive view towards evidence-based medicine and the access to the Internet for clinical decision making, practically of evidence-based medicine, and, they did not apply it since they did not consider a systematic training in this field [**18**].

The results of the research of Salehi and colleagues regarding the implementation of evidence-based performance on the nurses were different from the conclusions of this research. In titles of performance, most of staff in the field of evidence-based nursing was weak, the research staff was motivated, and the organization supported this weakness [**14**].

Unlike the model that predicted a causal relationship, there was no research that examined the related barriers and found no evidence of measures rejected by the customer orientation. With consideration of the measures used in this section, such as having enough time to find the evidence needed to have the confidence, having the necessary skills in the field of evidence-based practice, facilities, proficiency in English, it could be achieved that the presence of these barriers would be the best evidence that doctors can access. A large number of respondents and lack of time was the most significant factor in the judgment of not using evidence-based medicine. In 2004, Hanson and colleagues noted that only 1.9 percent of the physicians and other doctors use specific methods to find the evidence they need to learn to assess the evidence [**36**].

The previous researches noted other similar obstacles such as shortage of time, shortage of skills to search, the age of employees, level of experience, loss of a systematic training, their motivation and lack of an organizational support personnel [**11**,**13**,**17**,**19**].

Unlike the model, a causal relationship between practitioners in the field of evidence-based practice and customer orientation was observed and the fourth hypothesis was rejected. The study results also showed that the awareness of target groups used the low knowledge of employees in different job categories in this area [**2**,**8**-**16**].

It can be achieved that physicians studied the need for spending programs and training courses based on approach evidence, the signification of the work and being familiar with the advantages of using evidence-based approach. Therefore, it was recommended through workshops and educational meetings and the formulation of an interaction with the care centers based on evidence and past experience with regard to the facilities given to this important issue. Based on the findings of the fifth research hypothesis, the loss of barriers in the act of the greatest evidence was accepted by the customer. Based on the outcomes of Heiwe et al. study, it can be stated that despite some obstacles, like the lack of time, the approach based on evidence in the clinical practice, the judgment that the best evidence will be capable of taking better decisions and providing a greater quality of care was reached [**11**].

The sixth hypothesis, regarding the presence of a positive connection between the sources of evidence usually applied to retrieve the information on the customer, was accepted. The provision of the evidence resources needed such as manuals and instructions for entrance to the Internet, studying the data, training courses and intelligence information from colleagues shared a very significant role in the adoption of the evidence-based approach in the research group. The organization will provide the conditions and resources so that workers are directed towards the adoption of this approach. 

The outcomes of this research revealed that there is a meaningful causal connection between the dimensions such as “attitude”, “intent to conduct and behavior”, “lack of barriers to change based on the best evidence”, “the current evidence sources used for information retrieval”, and the evidence-based strategy to “customer” in the collection. But, there was no meaningful causal connection between the 2 dimensions, “lack of barriers and finding the evidence” and “Introduction to Evidence Based Practice”, the evidence-based strategy to “customer orientation” in the collection of physicians studied. By providing the necessary training so that the staff could provide the facilities to encourage and motivate the staff, the use of evidence-based program could be used and it would facilitate a greater efficiency and ultimately improve the organization and attract customers and clients. As physicians base their actions on the scientific evidence in the health area, they will be able to make better decisions and provide a higher level of care, so as to decrease the charge of treatment, patients gaining the satisfaction and the performance of the organization. The outcomes of this study showed that physicians make an evidence-based, customer-focused approach of the institution. 

**Recommendations**

In the end, it was suggested that similar studies in different working groups in the healthcare organization, were appropriate and compatible with the circumstances in our nation, the improvement of care being based on the achieved evidence-based practice.
